# The Physical Body Experiences Questionnaire Simplified for Active Aging (PBE-QAG)

**DOI:** 10.2174/1745017901814010070

**Published:** 2018-03-26

**Authors:** Giulia Cossu, Emilio Loi, Mauro Giovanni Carta, Alessia Bramanti

**Affiliations:** 1 Department of Medical Sciences and Public Health, University of Cagliari, Cagliari, Italy; 2 Istituto di Scienze Applicate e Sistemi Intelligenti, ISASI, Messina, Italy; 3 IRCCS Centro Neurolesi “Bonino-Pulejo”, Messina, Italy

**Keywords:** Active aging, Physical Awareness, Elderly, Physical Activity, Physical Body Experiences Questionnaire, International Physical Activity Questionnaire

## Abstract

**Background:**

The physical activity has been indicated as an experience that can help achieve positive, self-oriented own body awareness. This awareness is an aspect that tends to get worse with age.

**Objective:**

Our study aims to verify the internal consistency of a questionnaire on physical awareness in a sample of Italian elders; a secondary objective is to measure if there is a relationship between physical awareness and perceived level of physical activity.

**Methods:**

Cross sectional study on a consecutive sample of elderly people was administered the “Physical Body Experiences Questionnaire simplified for active aging (PBE-QAG)”, inspired by the “Physical Body Experiences Questionnaire”, modified, simplified and adapted to be used in the elderly over 65. To elderly people the International Physical Activity Questionnaire. Cronbach’s alpha was also used to assess internal reliability of the total PBE-QAG. The factor structure was evaluated through Confirmatory Factor Analyses (CFAs).

**Results:**

The Cronbach’s alpha was 0.8 for the “body-mind relationship” scale, 0.81 for the “accepting your body” scale, 0.83 for the “awareness of physical skills” scale, and 0.65 for the “awareness of physical limits” scale. Cronbach’s alpha for the total PBE-QAG was 0.89. The CFA indicated a model with the 4 factors (CFI = 0.989, TLI = 0.984, RMSEA = 0.076).

People who conducted physical activity assiduously or regularly and over 10 minutes showed a better score to the PBE-QAG than those who declared a sporadic activity and for “less than 10 minute”, respectively.

**Conclusion:**

Our study revealed that the PBE-QAG shows an excellent total internal consistency. In the Italian sample of elderly people the questionnaire shows the model with the 4 factors described in literature.

## 1 INTRODUCTION

By 2050, 22% worldwide and 35.9% of Italy population will be over 65 years. For many people, aging is characterized by the rise of chronic diseases. Thus, DALYs (Disability-Adjusted Life Years) will increase proportionally, with a dramatic burden on social and health costs [1]. This alarm was highlighted by the European Union, which has indicated the research on active aging as a priority [2].

There is evidence of significant benefits from physical activity (PA) and exercise in the psychosocial, and chronic disease-related disability [3].

PA, including exercise and sport, is associated with better immune responses in old age: it impacts on metabolic disorders, including diabetes [4]. A systematic review has shown that physical exercise prevents falls in older people [5] and lowers cognitive and memory decline [6]. We have conducted several studies on the use of physical exercise in the improvement of well-being, quality of life and depression symptoms, particularly in the elderly [7-12].

Among the dimensions that the physical activity expected to improve in the elderly there is the awareness of one’s body and a better acceptance of one’s own physicality, aspects that tend to get worse with age

Indeed, a recent study of Fredrickson and colleagues [13] has identified physical activity as an experience that can help achieve positive, self-oriented own body awareness. In this study, the authors introduced the concept of “embodiment” defined as the state in which a subject has cognition of his body as an essential and accepted part of his life; subsequently, this element has been seen as a potential protective factor against body image misperceptions and eating disorders According to Fredrickson and colleagues [13], the body awareness would consist of two components: “objectification of own body” and “subjective feelings of own body”. “Objectification of own body” occurs when a person takes cognition of how his/her body can be seen from the outside (“As I look at others?”), as opposed to the “subjective feeling of own body” in which a person is reflecting without outside comparison (“How do I feel with my body?”). Objectification theory [13] implies that there is a pressure dependent on culture adopting (endorsing) an observer perspective on own physical self. This self-objectification is required to lead to body shame and to use attention resources. The introspective awareness of own body (called non-objectivizing) is hypothesized to be more healthy because it sets people free being in harmony with the feelings of own body and allowing to appreciate the functionality of own physical self.

Our study aims to validate a questionnaire on physical awareness in a sample of Italian elders; a secondary objective of this study is to measure if there is a relationship between physical awareness and perceived level of physical activity.

We used a modified version of Physical Body Experiences (PBE) Questionnaire [14], which consists of 36 items. The questionnaire of Menzel [14] is not very suitable to be used in Italian elderly sample because it is too long and even the answers are not anchored to examples easily understood for an advanced Italian generation. It has never been validated in Italy or in the elderly population. The results of the study of Menzel [14] indicate that the PBE Questionnaire consists of four different dimensions, which refer to those aspects that the author calls the “embodiment”: mind-body relationship, acceptance of his own body, awareness of physical skills and awareness of physical limits [14].

The questionnaire used in this paper is an extremely simplified tool compared to the instrument that inspired it; moreover, it has been determined to be used in studies on elderly who use different outcome measures. The tool should, therefore, be as sustainable as possible.

## 2 MATERIALS AND METHODS

### 2.1 Study Design/Sample

The cross sectional study on a consecutive sample of elderly people was recruited at the University of the Third Age in the city of Cagliari, Italy.

### 2.2 Instruments

**A)** The “Physical Body Experiences Questionnaire simplified for active aging (PBE-QAG)”, has been inspired by the previously described instrument, PBE Questionnaire [14], but modified, simplified and adapted for the elderly over 65, in Italian language. It is shorter than the instrument that inspired it. This is a 12 item tool (see appendix) whose responses are on a 5-point Lickert scale (from 1 = “Totally True” to 5 = “Totally False”). The questionnaire presents three questions for each of the hypothetical 4-dimensional factors of physical awareness (body relationship, acceptance of one’s body, awareness of physical skills, awareness of physical limits), already identified by the previously cited study [11] and acknowledged as useful for the research in the elderly.

**B)** The International Physical Activity Questionnaire (IPAQ) [15] is a self-administered 9-item questionnaire that evaluates physical activity levels (as sedentary, light, moderate and vigorous) and energy expenditure over the past 7 days. The questionnaire was used to measure if the level of consciousness of the body, investigated by (PBE) Questionnaire also through the Confirmatory Factor Analyses (CFA), had a relationship with frequency, duration and intensity of physical activity reported by each elderly participant.

### 2.3 Statistical Analysis

The 12 items of the PBE-QAG were recorded and scored with those participants who received 1 mark for totally true and a 5 mark for indicating totally false. Responses from 2 to 4 indicated intermediate values.

Descriptive statistics were produced for the study participants, and included count and percentage. Observations with missing values have been excluded from the analyses. Cronbach’s alpha has been used to assess internal reliability of the PBE-QAG total and its sub-scales. The factor structure was evaluated by means of Confirmatory Factor Analyses (CFAs) performed using the estimator WLSMV that analyzes the polychoric correlation matrices, treating all the items as ordinal in the analyses. Goodness-of-fit indices were used to evaluate the models: Comparative Fit Index (CFI) and Tucker-Lewis Index (TLI) ≥.95 and ≥ .90 indicated good and sufficient fit, respectively. Root Mean Square Error of Approximation (RMSEA) ≤ .05 or ≤ .08 indicated good and sufficient fit, respectively. Factors’ correlation has been estimated through Pearson’s r coefficient. Factor indicators with loadings lower than .30 have been excluded from the analysis. Finally, path diagrams have been used for a visual comparison of the factor loadings.

To measure if the level of physical activity was associated with the level of physical awareness, a multivariate regression analysis has been used. The physical awareness level, measured as score the QCFA, represented the dependent variable. The level of physical activity (as item scores on frequency, duration, and intensity of the IPAQ) has been used as a dependent variable. The analysis has been conducted correcting gender, age and level of education (years of study) as possible confounding variables. All analyses have been performed with R 3.3.2.

## 3 RESULT

### 3.1 Descriptive Information

The study included 106 participants: 43 (40.56%) were men. Twenty-eight participants (24.52%) had at least 5-year of education, 30 (28.3%) 8 years and 31 (29.24%) 13 years of education, 13 (12.26%) had a degree and 4 (3.77%) a post-degree. Mean age was 68.88 years (53-95). Seven (6.6%) subjects were unoccupied, 12 (11.32%) were occupied and 81 (76.4%) were retired. The 12-item QCFA-total score mean was 25.16±9.58.

### 3.2 Internal Reliability of the PBE-QAG

The Cronbach’s alpha was 0.8 for the “body-mind relationship” scale, 0.81 for the “accepting your body” scale, 0.83 for the “awareness of physical skills” scale, and 0.65 for the “awareness of physical limits” scale. Cronbach’s alpha for the total PBE-QAG was 0.89.

### 3.3 Factor Structure

Factor structure has been evaluated to check the QCFA ability to measure the hypothesized four factor-dimensions of the questionnaire. We conducted a CFA, fitting a model with the 4 factors described in literature [13]. Overall, this model showed a good fit (CFI = 0.989, TLI = 0.984, RMSEA = 0.076). Fig. (**1**) reports the estimates of the loadings for the 4-factor model. All the items were associated with quite high loadings, even though it is worth noticing that the item 11 of the questionnaire on factor 4 was not high as the others (0.36).

Table **1** shows the strength of the association between physical awareness (QCFA score) and frequency of physical activity (IPAQ frequency of activity score); the measurement was correct for possible confounding factors. People who conducted physical activity assiduously (3/4 times a week) or regularly (1/2 times a week) showed a better score on the physical awareness questionnaire than those who declared a sporadic activity.

Table **2** shows the strength of the association between physical awareness (QCFA score) and duration of physical activity (IPAQ duration of activity score); the measurement was correct for possible confounding factors. People who conducted physical activity over 10 minutes (sum of “up to 20 minutes”, “up to 30 minutes”, “40 minutes or more”) showed a better score to the physical awareness questionnaire than those who declared “less than 10 minutes” of activity.

The perception of a different intensity of physical activity (IPAQ intensity score) did not seem to be associated with the level of physical awareness (QCFA score) (Table **3**).

## 4 DISCUSSION

The main result of our study is that the physical activities are associated with a better bodily awareness in elderly; this association, in turn, may be related to mental health benefits and to quality of life.

Our work revealed that the PBE-QAG shows an excellent total internal consistency measured through the Cronbach α coefficient. Indeed, the consistency is questionable when the coefficient is greater than 0.60; acceptable if is greater than 0.70; good when it exceeds 0.80; excellent when it exceeds 0.80 [16]. The internal consistency is also acceptable for the single sub-scales, even though the “Physical Limit Awareness” sub-scale has had a questionable performance at the limits of sufficiency. Factorial analysis confirmed the 4-factor structure (mind-body relationship; body acceptance; physical skills awareness; physical limitation awareness), which had been highlighted by a similar 36-item tool (PBE) [14], aimed to measure the same construct. In our study, we verified the scale validity to measure the construct in a specific sample of elderly people. Compared to the instrument previously validated in literature [14], PBE-QAG has the advantage of brevity, which makes it applicable also in studies in which multiple outcome needs to be taken into consideration and several instruments are required in relation to the different outcome measures which must be measured.

Especially, in studies on active aging, the awareness of own body is a measure that is interesting to take in consideration to quantify other dimensions of general well-being such as quality of life, satisfaction on social support or perception of social well-being. It may be also important to understand whether an impairment of this dimension may be related to the presence of psychopathological distress or disabling illnesses [17, 18]. In cohort studies, it is important to understand whether these dimensions can affect each other; in fact, physical awareness can be an editable construct [19].

The study confirms the conviction often reported in the literature (but rarely demonstrated) that a sufficiently sustained physical activity, as duration and frequency, is associated with better bodily awareness, although the study does not seem to confirm that a more intense physical activity increases the awareness. However, the data are preliminary. The hypothesis reveals a better physical awareness as a factor of well-being, which needs to be verified in longitudinal studies that may clarify the possible causal connection between physical activity and body consciousness. In relation to the demonstrated association, we can postulate that a greater physical activity produces better awareness and not vice versa.

To demonstrate the possibility to measure physical awareness and to confirm the dimensions of such construct in the elderly, as described in previous studies in non-elderly adults [14], this study can be considered as the first step to conduct other studies of this type on active aging.

The study has been carried out in a limited sample, whose recruitment methods (from a “University of the Third Age”) may have caused a bias of choice in relation to high schooling. However, it is important to note that in Italy the people are admitted to such “University of the Third Age” regardless of their previous educational level. In addition, the correction of the factor education in the analysis did not make any significant difference in the results.

The PBE-QAG, validate by larger studies, may be useful first in clinical trials and then in clinical practice, to evaluate how bodily awareness of elderly with chronic disease and its correlation with physical activity may impact on the quality of life and psychological well-being.

## CONCLUSION

Our study revealed that the PBE-QAG shows an excellent total internal consistency in elderly people. In addition, a sufficiently sustained physical activity was associated with better bodily awareness.

## Figures and Tables

**Fig. (1):**
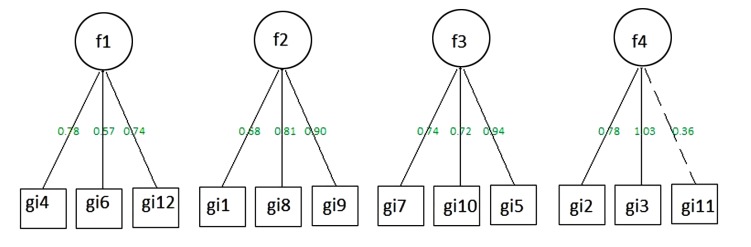
Stime of model of the 4 factors of QCFA.

**Table 1:** Results of the multivariate regression analysis with physical awareness level (measured as score the QCFA) as the dependent variable and frequency of physical activity (IPAQ frequency of activity score) as the independent variable (controlling for sex, age and education).

	Estimate	Standard Error	DF	t Value	P-value
**Intercept**	32.0030	15.4086	78	2.08	0.0411
**Frequency of physical activity**					
Assiduely (3-4 Times/Week)	-11.6363	3.1833	78	-3.66	0.0005
Regularly (1-2 Times/Week)	-7.3559	3.0768	78	-2.39	0.0192
Rarely (1-2 Times/Month)	-4.6847	3.5989	78	-1.30	0.1968
Never	0	.	.	.	.
**Gender**					
M	3.7293	2.2393	78	1.67	0.0998
F	0	.	.	.	.
**Age**	-0.05862	0.1940	78	-0.30	0.7633
**Education**					
Primary School	0	.	.	.	.
Middle School	3.7267	3.0520	78	1.22	0.2258
High School	3.3290	3.0903	78	1.08	0.2847
Graduation	0.5073	4.0547	78	0.13	0.9007
Post-Graduation	6.0574	5.5277	78	1.10	0.2765

**Table 2:** Results of the multivariate regression analysis with physical awareness level (measured as score the QCFA) as the dependent variable and activity duration as the independent variable (controlling for sex, age and education).

	Estimate	Standard Error	DF	t Value	P-value
**Intercept**	40.1698	15.3504	71	2.62	0.0108
**Duration of physical activity**					
Over 40 minutes	-13.2048	3.1416	71	-4.20	<.0001
Up to 30 minutes	-8.6276	3.9655	71	-2.18	0.0329
Up to 20 minutes	-11.4640	3.7561	71	-3.05	0.0032
Less than 10 minutes	0	.	.	.	.
**Gender**					
M	2.8436	2.1274	71	1.34	0.1856
F	0	.	.	.	.
**Age**	-0.1037	0.1890	71	-0.55	0.5850
**Education**					
Primary School	0	.	.	.	.
Middle School	1.0971	2.9029	71	0.38	0.7066
Higher Medium School	2.5322	2.9855	71	0.85	0.3992
Graduation	0.8287	3.9209	71	0.21	0.8332
Post-Graduation	4.6408	5.8946	71	0.79	0.4337

**Table 3:** Results of the multivariate regression analysis with physical awareness level (measured as score the QCFA) as the dependent variable and activity type as the independent variable (controlling for sex, age and education).

	Estimate	Standard Error	DF	t Value	P-value
**Intercept**	25.3261	17.5657	68	1.44	0.1540
**Kind of Physical activity**					
Quite Light	-3.2759	2.8419	68	-1.15	0.2531
Hard	-5.4430	5.6332	68	-0.97	0.3373
Quite Hard	-1.6440	3.7611	68	-0.44	0.6634
Light	0	.	.	.	.
**Gender**					
M	3.3285	2.4340	68	1.37	0.1760
F	0	.	.	.	.
**Age**	-0.01073	0.2218	68	-0.05	0.9616
**Education**					
Primary School	0	.	.	.	.
Middle School	-0.4590	3.3827	68	-0.14	0.8925
Higher Medium School	1.2184	3.5325	68	0.34	0.7312
Graduation	0.7414	4.6113	68	0.16	0.8727
Post-Graduation	7.8781	8.1403	68	0.97	0.3366

**Table:** Referring to my experience of the last 30 days.

	I can say that	Totally true				Totally false
1.	I don’t feel ashamed of my body at all (eg: I often wear clothes that enhance me, or I have no problem showing myself in a swimsuit at the sea or pool	1	2	3	4	5
2.	I avoid doing things that can expose me to the risk of hurting myself physically (eg: making excessive efforts, lifting weights too heavy for me)	1	2	3	4	5
3.	I am perfectly aware of my physical limits (eg: I know what kind of activities can be dangerous for my body)	1	2	3	4	5
4.	The physical activity I have being doing makes me feel satisfied and proud of myself.	1	2	3	4	5
5.	I trust that my body can learn new abilities (activities that I’ve never done before)	1	2	3	4	5
6.	Feeling in tune with my body makes me think that I’m effective and productive (eg: finishing activities I suppose to be possible for me makes me feel good)	1	2	3	4	5
7.	Doing vigorous physical activity can give me more strength and energy	1	2	3	4	5
8.	I feel aware of my physical abilities while respecting my imperfections	1	2	3	4	5
9.	I feel in harmony with my body (eg: I like taking care of my body and knowing what kind of things can be good for me or not)	1	2	3	4	5
10.	Mastering new physical abilities gives me great satisfaction (eg: doing activities I‘ve never done before)	1	2	3	4	5
11.	I do not feel comfortable pushing my body beyond its physical limits (eg: doing activities I suppose can be beyond my reach)	1	2	3	4	5
12.	I feel that the clarity of my thoughts depends on my physical well-being and my energy (eg: I find that by doing regular physical activity I'm mentally more active)	1	2	3	4	5

## APPENDIX

People can experience themselves during their daily physical activity in many different ways. We ask you to read the sentences below and indicate to what extent each statement is true for you. We ask you to evaluate it in the most honest and accurate way possible based on your personal experience of the last month.
